# Controlling the Molecular Structure and Physical Properties of Artificial Honeybee Silk by Heating or by Immersion in Solvents

**DOI:** 10.1371/journal.pone.0052308

**Published:** 2012-12-21

**Authors:** Mickey G. Huson, Jeffrey S. Church, Jacinta M. Poole, Sarah Weisman, Alagacone Sriskantha, Andrew C. Warden, Peter M. Campbell, John A. M. Ramshaw, Tara D. Sutherland

**Affiliations:** 1 Commonwealth Scientific and Industrial Research Organisation (CSIRO), Materials Science and Engineering, Geelong, Australia; 2 CSIRO, Materials Science and Engineering, Melbourne, Australia; 3 CSIRO, Ecosystem Sciences, Canberra, Australia; Max Planck Institute for Polymer Research, Germany

## Abstract

Honeybee larvae produce silken cocoons that provide mechanical stability to the hive. The silk proteins are small and non-repetitive and therefore can be produced at large scale by fermentation in *E. coli*. The recombinant proteins can be fabricated into a range of forms; however the resultant material is soluble in water and requires a post production stabilizing treatment. In this study, we describe the structural and mechanical properties of sponges fabricated from artificial honeybee silk proteins that have been stabilized in aqueous methanol baths or by dry heating. Aqueous methanol treatment induces formation of ß-sheets, with the amount of ß-sheet dictated by methanol concentration. Formation of ß-sheets renders sponges insoluble in water and generates a reversibly compressible material. Dry heat treatments at 190°C produce a water insoluble material, that is stiffer than the methanol treated equivalent but without significant secondary structural changes. Honeybee silk proteins are particularly high in Lys, Ser, Thr, Glu and Asp. The properties of the heat treated material are attributed to generation of lysinoalanine, amide (isopeptide) and/or ester covalent cross-links. The unique ability to stabilize material by controlling secondary structure rearrangement and covalent cross-linking allows us to design recombinant silk materials with a wide range of properties.

## Introduction

Silk-based materials are of interest for many applications, with over 300 patents around silk protein materials approved in the past five years. The silk produced by honeybees shares many desirable properties with the better known silkworm or spider silks, but has a more varied amino acid composition giving it a different surface chemistry and a niche for different applications [1]. Natural honeybee silk is composed of four related fibrous proteins [1] which assemble into a coiled coil structure [2]. Honeybee silk proteins can be produced recombinantly in *E. coli* [3], [4], and at high yield [4]. Solutions of single or all four recombinant honeybee silk proteins can be manufactured into material forms including fibers and films [4], [5], electrospun mats [6] and sponges (this work). All forms require post fabrication treatment of some description to stabilize the material to render them stable in aqueous solutions.

Stabilization of honeybee silk protein has been achieved after inducing a partial molecular structural transition from coiled coil to β-sheet structure by immersion in 90% methanol solution [4], immersion in 70% methanol solution [5] or by water annealing [6], presumably by generating β-sheet cross-links between the coiled coil proteins. β-sheet structure in coiled coil silk materials has also been induced by dry heating to 215°C, well above the protein's glass transition temperature [5], or by autoclaving at 121°C [7]. Similar approaches have previously been used for stabilization of regenerated silkworm fibroin material [8], [9], [10], [11], [12].

Methanol induces formation of β-sheets by increasing the hydrophobicity of the solvent and therefore weakening internal protein-protein hydrophobic interactions. At the same time it decreases the availability of water for hydrogen bonding thereby driving protein-protein hydrogen bonding [13]. In mixed water-alcohol systems the underlying mechanisms are much more complex with several distinct physicochemical effects impacting such transitions [14]. The structural transition of silkworm fibroin to β-sheet structure is rapid, and more complete in aqueous methanol solutions than in dry methanol solutions [12]. Methanol treatment of reconstituted silkworm silk increases the compressive strength of silkworm fibroin sponges, but the rapid dehydration induced also makes them brittle [11]. Changes to the mechanical properties of recombinant honeybee silk materials due to methanol treatment have not been previously investigated.

Collagen has been used extensively as a biomedical material. Various chemical and physical treatments have been used to stabilize collagen, giving insolubility and durability. For example, stabilization of collagen can be achieved by dry heat treatments that involve heating the material under vacuum to temperatures of 100–120°C for several days [15]. The stability of the treated material is due to the covalent cross-linking of the proteins rather than secondary structure protein changes. The major covalent cross-links found are amides formed by condensation reactions between amine and carboxylic acid side groups [15]. A small amount of lysinoalanine formed by dehydration of serine (or desulfation of cysteine) followed by reaction with lysine, is also present [16]. Heat treatment significantly increases the strength, extensibility and elasticity of regenerated collagen fibers [17].

This work systematically studies the effects of different stabilization treatments of artificial honeybee silk on the material's protein structure and mechanical properties. The two treatment types studied are submersion in aqueous methanol of varying concentrations and dry heat treatment using varying temperatures and times. The honeybee material form chosen for study is freeze-dried sponges, as these have a high surface-to-volume ratio that allows good penetration of solvents and can be manufactured at physical sizes large enough to allow simple and robust mechanical property measurements.

## Materials and Methods

### Generation of protein sponges

A honeybee silk protein (AmelF3; NCBI accession no: NP_001129680) was expressed into *Escherichia coli (E. Coli)* inclusion bodies and purified as previously described [4]. Briefly, purified inclusion bodies were solubilised in 3% aqueous sodium dodecyl sulfate (SDS) solution then treated with 300 mM KCl. The potassium serves to precipitate the dodecyl sulfate of SDS which can then be removed by centrifugation, thereby reducing SDS levels to around 0.3% (weight/volume) and producing high-purity AmelF3 solution [4]. The AmelF3 solution was dialysed against 15% polyethylene glycol (PEG), 0.25% SDS to remove salt and generate a solution containing 3.6% protein, 0.3% SDS. The solution also contained 0.1% salt comprising residual Na^+^ derived from the SDS after precipitation of the dodecyl sulfate and K^+^ with Cl^−^ counter ions. SDS concentration was determined according to Rusconi *et al.* [18], salt concentration was determined by calculation of dilution during dialysis, and total solute weight was determined by weighing aliquots of dried solution. The solution was poured into silicone rubber moulds (14×5×6 mm; RL060, ProSciTech, QLD), frozen at −20°C overnight, and placed in a freeze-dryer (FD355DMP, FTS Systems) for 24 hours to generate sponges with typical dimensions of 12.6×4.5×5.4 mm. The silk sponges were stored in sealed plastic bags at room temperature until use.

For comparison, stabilized collagen sponges were made from ovine skin collagen. Minced ovine skin was digested with 1 mg/mL pepsin at 4°C for 24 hours in 100 mM acetic acid adjusted to pH 2.5 with HCl. The hydrolysate was purified using two 0.7 M NaCl precipitation steps [19]. SDS-PAGE showed that the collagen was predominantly type I, but a small quantity of type III collagen, <5%, was also present. PEG 4000 was used to precipitate collagen as fibre-like aggregates [20] as previously described [21]. Purified collagen was dissolved at 2 mg/mL in 20 mM acetic acid then adjusted to pH 7.0 with 40 mM sodium phosphate buffer. PEG (40% w/v) was added slowly with stirring to a final concentration of 4 dry wt% solution and the solution was left at 4°C for 16 hours. The precipitate was collected as a loose pellet by centrifugation at 1000×g for 15 min then re-suspended in 20 mM sodium phosphate buffer, pH 7.0, and the precipitate re-collected as above after 1 hour. The precipitate was re-suspended in 10 mM sodium phosphate buffer, pH 7.0, and the precipitate re-collected after 1 hour at 1000×g for 30 minutes. The collagen paste was adjusted to 4% protein, transferred into silicone rubber moulds (RL060, ProSciTech, QLD) and freeze-dried. Collagen sponges were suspended in dry ethanol and air in the pores was removed by vacuum prior to cross-linking with a 10∶1 w/w excess of 1-ethyl-3-[3-dimethylaminopropyl]carbodiimide hydrochloride for 16 h at room temperature. The sponges were then washed extensively in ethanol and air dried.

### Stabilization of honeybee silk sponges and water solubility testing

Honeybee silk sponges were treated to decrease water sensitivity by either heating in air to temperatures of 160–190°C for times ranging between 10–120 minutes, or by immersion for 48 hours in aqueous methanol solutions, with methanol concentrations ranging from 0–100%. In order to test water stability, sponges were cut into 4 pieces and each sample, under ambient laboratory conditions, was weighed on a microbalance prior to heat or methanol treatment (initial mass). After methanol treatment, sponge samples were transferred through a dilution series of 40% and 20% methanol into water. All samples were incubated individually in 1 mL water at room temperature on an orbital shaker for time periods ranging from 1 to 32 days, dried at 60°C for 2 hours and then weighed to determine mass loss. Mass loss is reported as the percentage of the initial mass. Initial mass loss and rate of mass loss were determined from the y-intercept and slope of the linear regression through the mass loss vs. time data points, respectively. Four samples per treatment were assessed and data collected at 0, 1, 2, 4, 8, 16 and 32 days. SDS levels in the methanol solutions or water after sponges were removed were calculated according to Rusconi *et al.* [18], and protein concentration was determined using the QuantiPro BCA assay kit (Sigma; St Louis, MO).

### Compression testing

The mechanical properties of honeybee silk sponges and stabilized collagen sponges were measured using compression tests on an Instron 5500R (Instron, USA) fitted with a 2.5 N static load cell. Methanol treated samples were treated in 60–100% methanol in water for 48 hours and then transferred through a dilution series of 80, 60, 40 and 20% methanol solutions each for 10 minutes and then soaked for 24 hours in phosphate buffered saline (PBS) prior to testing. Heat treated samples and collagen sponges were soaked for 5 minutes in PBS with gentle squeezing to ensure saturation prior to testing. Sponge samples were compressed to 190–200 mN at 2 mm/min then decompressed at 2 mm/min. The compression tests were repeated three times on each sample with 1 minute relaxation time between tests. The cross-sectional area of the samples, measured on a light microscope to be around 57 mm^2^, was used to convert force values to stress values. Sample thickness was determined during the compression test, as the distance between the base plate and the point at which the compression plate contacts the sample (F = 1 mN).

### Raman spectroscopy

Raman spectra were obtained from dried honeybee silk sponges at a resolution of 4 cm^−1^ using a Bruker RFS-100 FT-Raman spectrometer (Karlsruhe, Germany) equipped with an Adlas Nd:YAG laser operating at 1.064 µm and 500 mW and with a liquid nitrogen cooled Germanium diode detector. Spectra were collected using 180° backscatter geometry with the samples held in a compression cell described elsewhere [22]. Data acquisition over 512 scans was performed using Bruker OPUS software (version 3.1). Four spectra were obtained from different areas of each sample and averaged to produce a final spectrum for analysis. All spectra obtained from a given sample were found to be in excellent agreement. Data manipulation was carried out using Grams AI v8.0 software (Thermo Electron Corp., USA). All spectra were normalized on the C–H bending mode at 1449 cm^−1^ as this mode is insensitive to protein conformation. The structural changes of the honeybee silk sponges after the different treatments was also assessed by the quantitation of the overlap area between the second derivatives of the amide I bands [23]. Briefly, the method, which was implemented using Matlab R2010a (Math Works, USA), involves the normalization of the spectra between 1620 and 1730 cm^−1^ followed by the calculation of their 2^nd^ derivatives using the method of Savitzky and Golay [24]. The two 2^nd^ derivative spectra to be compared were passed through a logical filter which takes the lowest intensity value of the pair and creates an array that constitutes a spectrum representing the overlap area.

### Amino acid analysis

Quantitative amino acid analysis *via* acid hydrolysis was carried out on untreated and heat treated honeybee silk sponge samples. Digestions were carried out in triplicate, analyzed in duplicate and the results reported as an average. Deficits of amino acids in the treated samples relative to untreated samples were taken to indicate residues that had been modified by heat-treatment.

Modifications such as dehydration reactions producing isopeptide bonds would be reversed by acid hydrolysis. In order to identify residues involved in such modifications, amino acid analyses were also performed following enzymatic digestions of silk sponge samples. Enzymatic digestions were performed in duplicate on samples of heat-treated and non-heat-treated honeybee silk sponges that were first washed with 70% methanol. Methanol would not be expected to induce any covalent modification but removes detergent that might interfere with enzymatic digestion or mass spectral analysis (below).

Approximately 3 mg of sample was accurately weighed and resuspended in 100 mM HEPES buffer pH 7.5. The samples underwent a 24 hour enzymatic hydrolysis at 37°C with Pronase E, Leucine Aminopeptidase M, and Prolidase in a final volume of 500 µL. Samples were heated at 100°C for 10 minutes before undergoing a further 24 hour enzymatic hydrolysis at 37°C with carboxypeptidase.

Levels of amino acids after both treatments were analyzed using the Waters AccQTag Ultra chemistry on a Waters ACQUITY UPLC system. The non-standard amino acid ε-(γ-glutamyl)-lysine was quantified by reference to a standard (Sigma) and eluted with a retention time similar to cysteine, an amino acid that is conveniently absent from AmelF3.

### Liquid chromatography-tandem mass spectrometry

Targeted liquid chromatography-tandem mass spectrometry (LC-MS/MS) was used to test for the presence of ε-(γ-glutamyl)-lysine, a dipeptide that would remain following enzymatic digestion of honeybee silk sponges containing Glu-Lys iso-peptide crosslinks. The method is based on a published method [25], showing tandem mass spectra that distinguish the isolated isopeptide crosslink from isomeric dipeptides of lysine and glutamate. Heat-treated and non-heat-treated material was digested as above, acidified with formic acid (1% v/v final) and 1 µl was separated on an Agilent 1200 capillary LC system coupled by electrospray ionization to an Agilent XCT ion trap mass spectrometer. The column (Agilent Zorbax 300SB-C18, 3.5 µm, 50×0.3 mm) was eluted isocratically with 3% acetonitrile/0.1% formic acid at 5 µL/min for 5 minutes before washing with 90% acetonitrile/0.1% formic acid and re-equilibration with 3% acetonitrile/0.1% formic acid. The ion trap was tuned to retain only ions of 276 *m/z*, corresponding to the MH^+^ ion from ε-(γ-glutamyl)-lysine. Fragmentation energy was adjusted to produce two dominant ions at 131 and 148 *m/z* from a standard solution of ε-(γ-glutamyl)-lysine (Sigma) to obtain spectra consistent with that previously reported [25]. The standard eluted at 1–2 minutes. Mass spectra from the same retention time were averaged for digested honeybee silk sponges for comparison with the spectrum from the iso-peptide standard.

## Results and Discussion

### Efficiency of stabilization treatments

The efficiency of stabilization treatments on recombinant honeybee silk materials could be assessed by the performance of the material in water, with untreated sponges swelling and rapidly dissolving. Sponges that were treated either by heating dry material for various times or by immersion in aqueous methanol solutions were incubated in water for up to 32 days and their water solubility monitored (Table 1). Treatment with aqueous solutions containing >50% methanol but <100% methanol, or heat treatment at 190°C, imparted a high degree of water stability to the sponges.

**Table 1:** Stability of honeybee silk sponges in water over 32 days. Standard deviations are given in brackets, ns = not significant at the 95% confidence limit.

Treatment to cross-link proteins	Initial mass loss (%)	Average further mass loss per day (%)	Degree of swelling
Untreated	100	-	-
48 h in 60% MeOH	18 (2)	0.0 (ns)	Low
48 h in 80% MeOH	23 (2)	0.0 (ns)	Med
48 h in 100% MeOH	21 (8)	1.0 (0.4)	High
10 min @ 160°C	100	-	-
10 min @ 180°C	100	-	-
10 min @ 190°C	18 (2)	0.3 (0.2)	Med
30 min @ 190°C	17 (2)	0.0 (ns)	Low
60 min @ 190°C	18 (2)	0.0 (ns)	Low
120 min @ 190°C	19 (2)	0.0 (ns)	Low

When a sponge was subjected to its initial wet exposure, either as part of its treatment and/or during the first day of water stability testing, a mass loss of 18–23% occurred (Table 1). This loss was primarily due to leaching of SDS from the sponges. The detergent was detected in the methanol solutions or water at levels equivalent to what was originally in the sponge (data not shown). The remaining mass lost is likely to be made up of leached salts and low molecular weight peptides present as contaminants or degradation products, and bound water loss during the dry heat treatment process (4–6%) or resulting from molecular rearrangement.

Samples treated with aqueous methanol solutions containing <50% methanol dissolved in the treatment solutions. Sponges stabilized for up to 48 hours in solutions containing very low levels of water (100% methanol treatment), swelled extensively when transferred to water and continually lost protein at a rate of around 1% per day (Table 1). The swollen sponges were too weak to be lifted out of the solution intact and hence could not undergo mechanical testing. The water stability of the swollen sponges, as determined after the sponge fragments were collected by centrifugation and weighing, was significantly greater than the untreated material (Table 1) despite their mechanical weakness.

Samples that had been heat treated at temperatures <190°C (160°C, 180°C) were significantly less water stable than those treated for equivalent times at 190°C, the samples treated for 10 minutes dissolving completely in the first 24 hours of incubation (Table 1). Samples heated for 10 minutes at 190°C continually lost protein, at a rate of around 0.3% per day. By contrast the samples heated for longer time periods at 190°C were effectively impervious to water after their initial mass loss (Table 1).

### Effect of aqueous methanol stabilization on protein molecular structure

**Figure 1:**
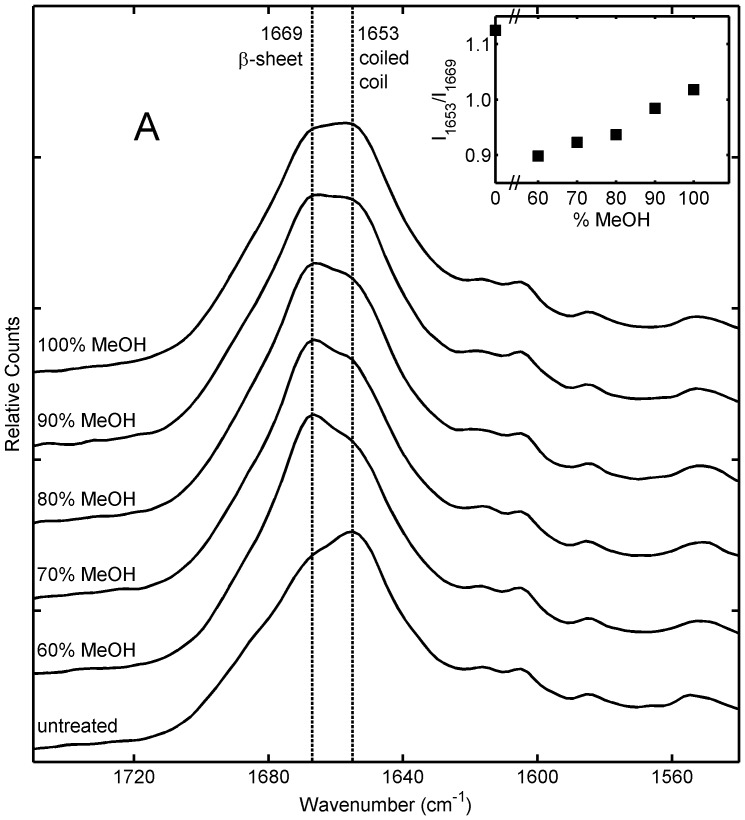
Raman spectra of honeybee silk sponges immersed for 48 hours in aqueous methanol solutions containing 60–100% methanol. Inset shows the approximate ratio of Raman intensity at 1653 cm^−1^ (attributed to coiled coil protein structure) to intensity at 1669 cm^−1^ (attributed to ß-sheet protein structure) for different samples.

Raman spectroscopy is a well established technique for assessing changes in protein conformation [26], [27], [28], with the semi-quantitative estimation of protein conformation by Raman spectroscopy in good agreement with those determined by X-ray diffraction and infrared spectroscopy [28]. Raman spectra of aqueous methanol treated sponges obtained immediately after treatment are shown in Figure 1. Spectra obtained from sponges that had been soaked in water for 24 hours after aqueous methanol then dried and analyzed were effectively identical to the spectra collected from dry sponges immediately after treatment, indicating that water immersion did not affect the protein secondary structure after stabilization (data not shown). Examination of the amide I region of the spectra indicated a distinct change in protein conformation as a consequence of the aqueous methanol treatment (Fig. 1). There is an increase in the peak at 1666 cm^−1^, attributed to ß-sheet, and a corresponding decrease in the peak at 1655 cm^−1^, attributed to coiled coil structure [5], [27], [29]. As it is not possible to resolve ß-sheet and disordered protein structure in the amide I region [29] there is a possibility that both are actually present. In the amide III region (not shown) where the ß-sheet and disordered proteins are better resolved, there is a slight increase in a feature at 1258 cm^−1^ confirming that a small level of disordered protein is being formed [29], particularly at the lower methanol concentrations. There is a trend towards decreasing structural change with increasing methanol concentration (Fig. 1, inset), so that the sample treated with 60% methanol has the highest ß-sheet content (corresponding with the lowest level of water swelling in Table 1), and the sample treated with 100% methanol has the lowest ß-sheet content (corresponding with highest level water swelling and some water solubility in Table 1).

**Figure 2:**
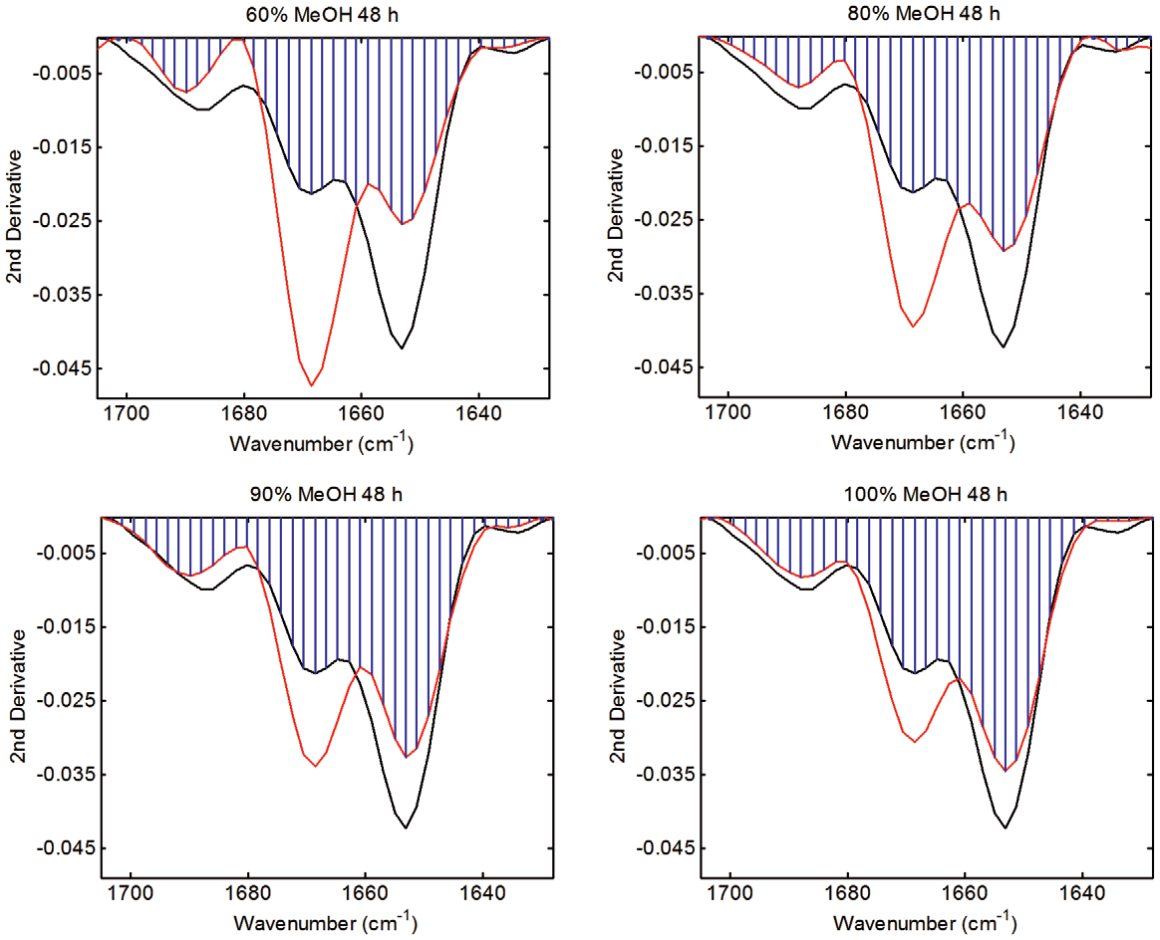
Amide I 2^nd^ derivative band overlap analysis of honeybee sponges treated with aqueous solutions of 60, 80 or 90 methanol or 100% methanol for 48 hours. Untreated sponge (black trace), treated sponge (red trace) and overlap area (blue shading).

The structural change upon aqueous methanol treatment was further investigated using the band overlap approach developed by Kendrick *et al*. [23] for the analysis of amide I bands (Table 2, Fig. 2). The band overlap analysis provides a single measure of difference and is ideal for assessing minor conformational changes in a series of samples, and thus was used in preference to the more traditional full band deconvolution method [26], [27], [28]. Band overlap analysis confirmed that the greatest conformational change (26%) was observed for the 60% methanol treated sponge while the least (11%) was found for the 100% methanol treated sponge. The percentage conformational change was found to be a linear function of methanol concentration with an r^2^ of 0.9838. The changes consisted of a major loss in coiled coil structure (1653 cm^−1^) off-set by a major gain in ß-sheet structure (1669 cm^−1^). There was also a minor loss in ß-turn structure observed at 1688 cm^−1^ [27].

**Table 2:** Conformational change of recombinant honeybee silk materials as a function of stabilization treatment.

Treatment	Conformational Change (%)	Description
60% MeOH 48 h	26	Major loss of coiled coil structure (1653 cm^−1^) off-set by a major gain in ß-sheet structure (1669 cm^−1^). There is also a minor loss in ß-turn structure observed at 1688 cm^−1^
70% MeOH 48 h	24	
80% MeOH 48 h	19	
90% MeOH 48 h	14	
100% MeOH 48 h	11	
160°C 60 min	6	
180°C 60 min	6	Loss of coiled coil structure (1653 cm^−1^) coupled with gain in ß-turn structure (1688 cm^−1^)
190°C 10 min	5	
190°C 30 min	10	Greater loss of coiled coil structure (1653 cm^−1^) coupled with gains in ß-sheet and turn structures at 1669 and 1688 cm^−1^
190°C 60 min	12	
190°C 120 min	10	No significant further loss of coiled coil structure (1653 cm^−1^) but no apparent gain in ß-sheet structure at 1669 cm^−1^

In aqueous methanol treatments, water acts as a plasticizer, lowering the glass transition temperature of the protein and thus increasing the mobility of the protein and allowing structural rearrangement to occur more readily. Since water is also a solvent for the protein, stabilization of the material requires a compromise to be reached; too much water and the dissolution process dominates, too little or no water and the rate of structural rearrangement becomes unacceptably slow. Thus sponges treated in less than 50% methanol dissolved in the high aqueous environment. Sponges treated in 50–90% methanol were in an environment with sufficient water to allow protein structural rearrangement but also sufficient methanol to keep the protein precipitated and in the solid form. In the absence of water (100% methanol treatment), the sponges did not undergo sufficient protein rearrangement during the treatment period (48 hours) to generate a stable material and the material swelled extensively when transferred to water (Table 1).

### Effect of dry heat stabilization on protein molecular structure

**Figure 3:**
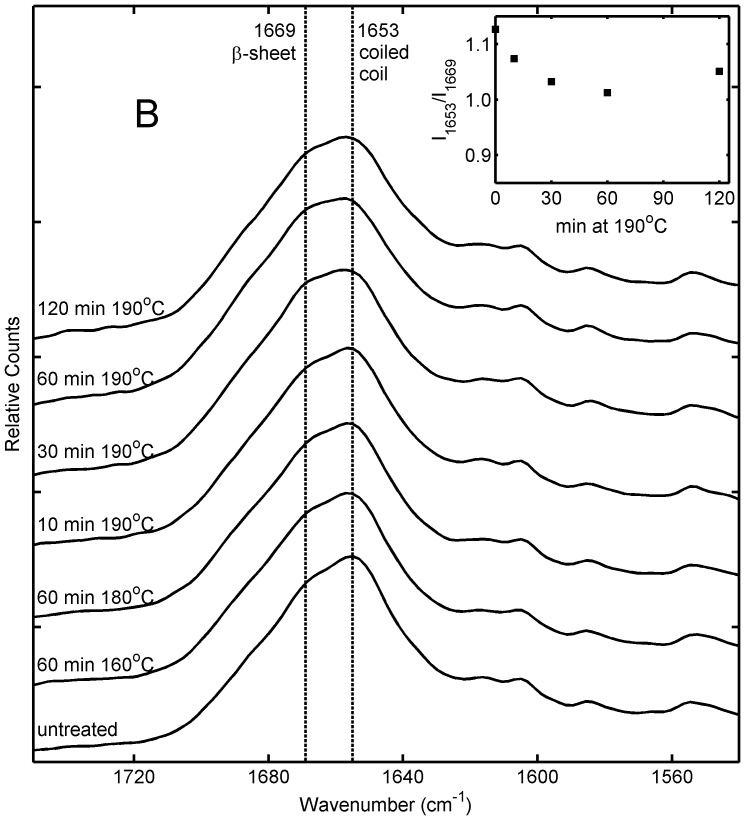
Raman spectra of honeybee silk sponges treated by heating at different temperatures for periods of 10–120 min. Inset shows the approximate ratio of Raman intensity at 1653 cm−1 (attributed to coiled coil protein structure) to intensity at 1669 cm−1 (attributed to ß-sheet protein structure) for different samples.

Previously, it had been shown that dry heating of artificial honeybee silk protein films to 215°C led to substantial ß-sheet formation [5]. In this study, the Raman spectra of sponges heated to 190°C remained similar to that of untreated sponges with only a slight structural transition in the direction of ß-sheet indicated in the amide I region (Fig. 3) and no significant differences observed in the amide III region. In the time series of samples treated at 190°C, the effect is smallest in the sample treated for 10 minutes then approximately constant for treatments of 30 minutes or longer (Fig. 3, insert). In all heat treated sponges the magnitude of the structural transition is less than that seen in samples treated with 100% methanol. However, the heat treated samples have high water stability, significantly better than sponges treated with 100% methanol (Table 1), suggesting that the heat treated samples are undergoing stabilizing changes other than the dramatic protein structural transitions observed in methanol treated samples.

**Figure 4:**
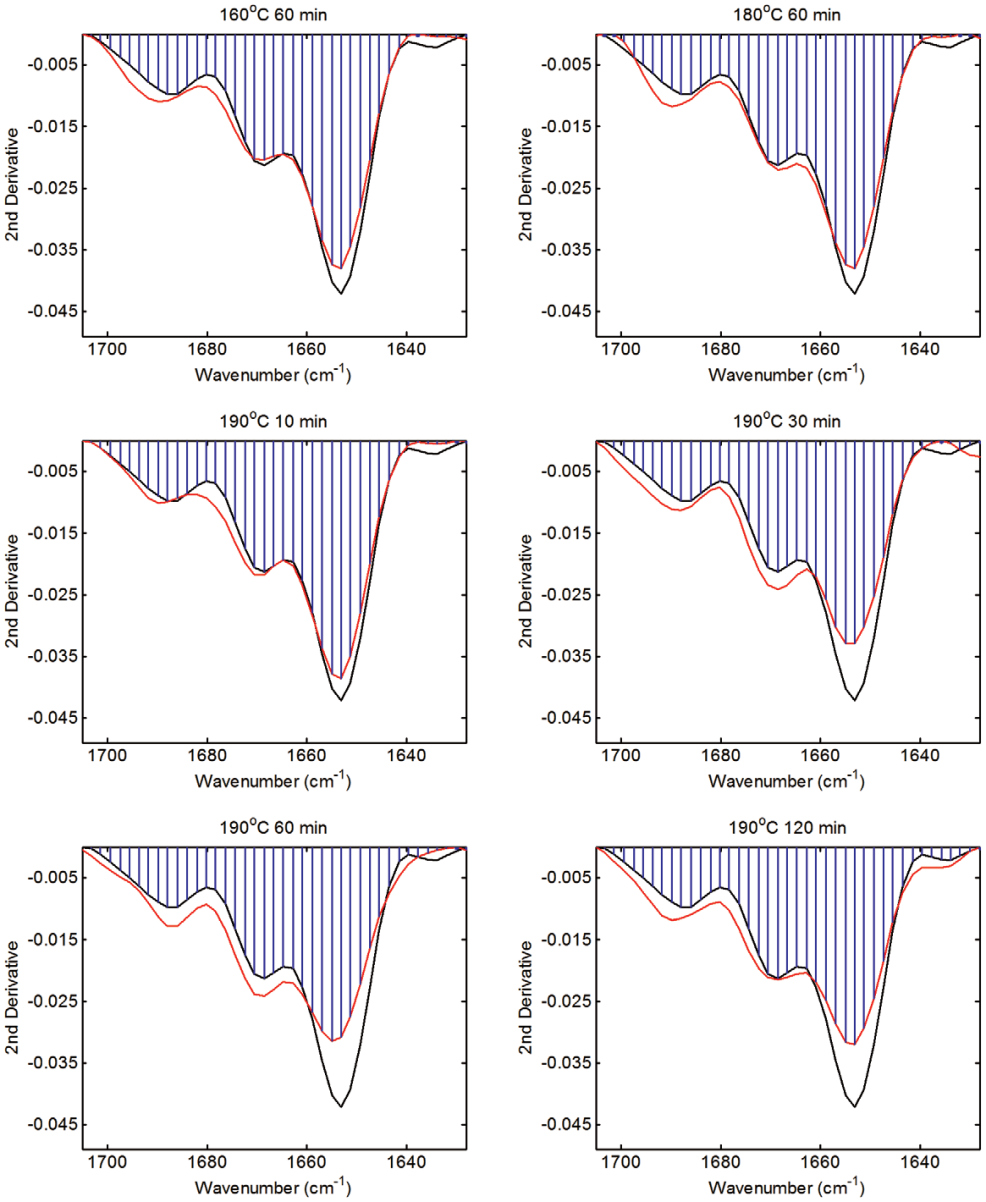
Amide I 2^nd^ derivative band overlap analysis of honeybee sponges heat treated at 160, 180 and 190°C for different lengths of time. Untreated sponge (black trace), treated sponge (red trace) and overlap area (blue region).

Amide I band overlap analysis results are presented in Table 2 and Figure 4. The conformational similarity of the sponges treated at 160 and 180°C for 60 minutes and 190°C for 10 minutes showed all underwent structural rearrangements of the order of 6% upon treatment. These changes can be associated with the loss of coiled coil structure coupled with gains in ß-sheet structure. Sponges treated at 190°C for more than 30 minutes also have very similar conformations to each other, having undergone conformational change of the order of 10%. These sponges exhibit greater loss of coiled coil structure along with complementary gains in ß-sheet structure compared to those treated at lower temperature or for less time.

### Mechanical properties of treated sponges

**Figure 5:**
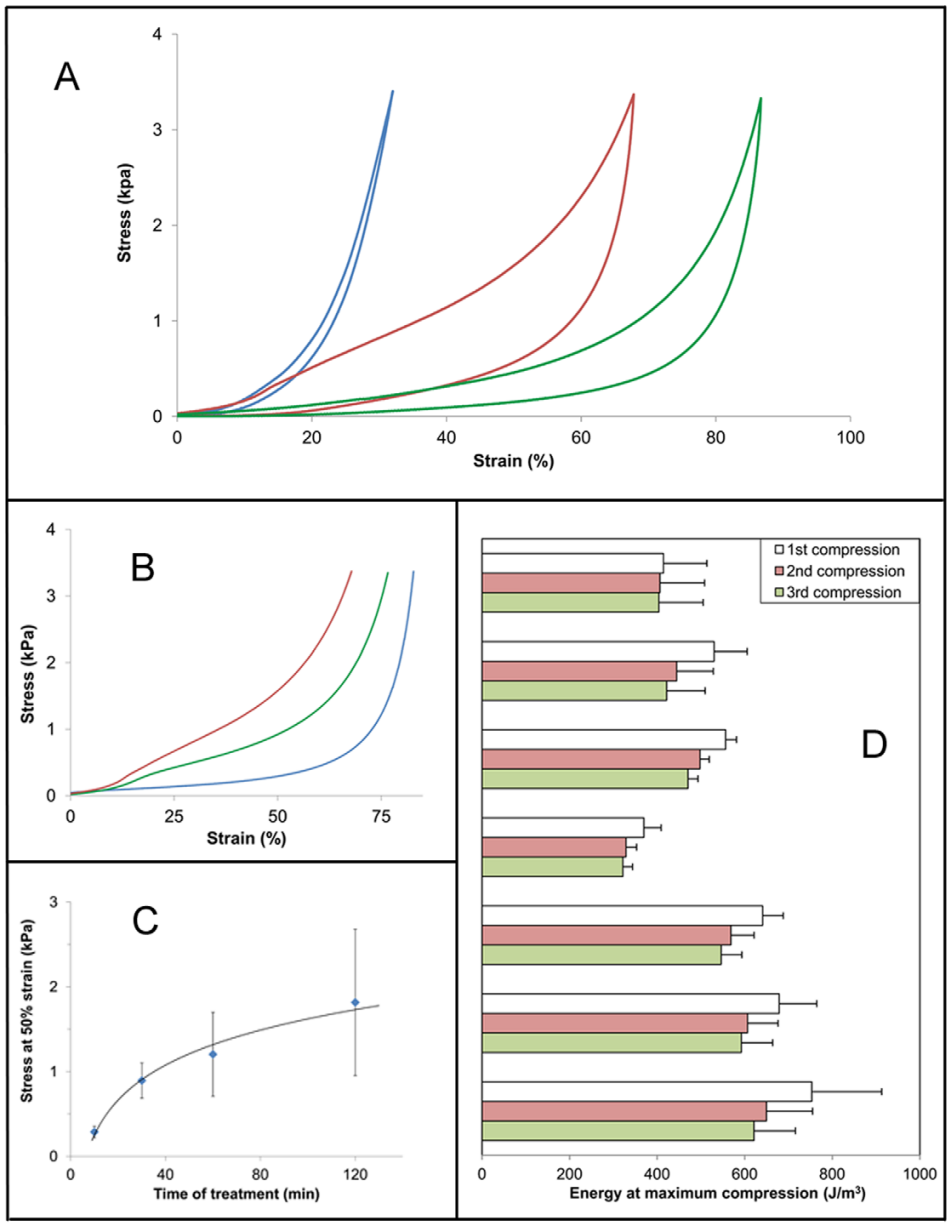
Compressive mechanical properties of sponges. A) Representative 1^st^ cyclic compression curves from collagen (green) and honey bee silk stabilized by heat treating for 120 min at 190°C (brown) or by immersion in 80% methanol for 48 hours (blue), B) Representative 1^st^ compression curves of honey bee silk sponges stabilized by heat treating at 190°C for 10 min (orange), 60 min (purple) or 120 min (brown). C) Change in stiffness of honey bee silk sponges as a function of time of heat treatment at 190°C, D) Energy required to compress the sponges to 0.2 N, with three compression cycles separated by one minute relaxation times. Error bars represent standard deviations.

Honeybee silk sponges treated by heating or by immersion in aqueous methanol solutions were subjected to compression tests in phosphate buffered saline (PBS) solution to assess their wet mechanical properties. For comparison, sponges of collagen, an established biomedical material, chemically cross-linked to a level that allows the sponge to return to its original size, were also tested. Representative compression curves are shown in Figure 5A and summarized results are shown in Table 3. Since the sponges have been compressed to a constant force, the compressive strain at this load is inversely related to the stiffness of the samples. The best measure of stiffness, however, is the stress at a given strain. Clearly, the honeybee silk sponges are softer than the collagen sponges requiring less stress to compress the sponge to 25% of its initial thickness (i.e. at 25% strain, Table 3). There is no significant difference between the honeybee samples treated in 60% or 80% methanol, both of which are softer than the samples heated at 190°C for greater than 10 minutes (compare stress at 25% and 50% strain). The energy required to compress the sponges is influenced by both the stiffness and shape of the curve and so is best used only as an indicator of the recovery of the samples between successive compressions (Table 3 and Fig. 5D). The samples that were heat treated show an initial J-shaped compression curve after 10 minutes heating (Fig. 5B). At longer heat treatment times a small yield point is evident (Fig. 5B) and the sample stiffness (stress at 50% strain) and energy required for compression increase logarithmically with time of treatment (Fig. 5C and Table 3).

**Table 3:** Average values of the mechanical properties of sponges subjected to cyclic compression testing in PBS.

Sample	Treatment to cross-link proteins	Strain at max load (%)	Stress at 25% strain (kPa)	Stress at 50% strain (kPa)	Energy to max load (Jm^−3^)	Resilience (%)
Collagen	carbodiimide	39 (11)	1.43 (0.89)	-	414 (99)	83 (2)
AmelF3	48 h in 60% MeOH	93 (3)	0.14 (0.05)	0.38 (0.11)	530 (75)	41 (2)
AmelF3	48 h in 80% MeOH	90 (2)	0.20 (0.04)	0.44 (0.04)	556 (25)	46 (2)
AmelF3	10 min @ 190°C	83 (1)	0.13 (0.03)	0.29 (0.07)	370 (39)	45 (2)
AmelF3	30 min @ 190°C	76 (2)	0.41 (0.06)	0.89 (0.21)	641 (47)	53 (1)
AmelF3	60 min @ 190°C	72 (6)	0.52 (0.22)	1.20 (0.49)	679 (86)	48 (4)
AmelF3	120 min @ 190°C	65 (8)	0.81 (0.49)	1.82 (0.86)	753 (159)	38 (4)
AmelF3 *	120 min @ 190°C	84 (7)	0.33 (0.03)	0.97 (0.40)	760 (140)	55 (2)

All samples were compressed to a maximum load of 0.2 N. All values are for the first cycle except the sample marked by the asterisk (*) which is for the first cycle of the 3^rd^ compression experiment after 32 days in PBS. Values in brackets are standard deviations.

Resilience is measured as the ratio of the energy required to deform a sample, to the energy recovered when the load is removed. In other words, it is a measure of a material's ability to recover from deformation. Resilience values of the stabilized honeybee silks vary between 40 and 50%, substantially lower than the 80% shown by collagen. It should be noted, however, that the collagen sample, being much stiffer, was only compressed to about 40% compared to 70–90% for the honeybee silk samples. As a general rule, resilience decreases with increasing energy of deformation [30], therefore comparing samples which require vastly different energies of deformation can be misleading. It is important to note too, that the values in Table 3 are a measure of the instant recovery during the cyclic test and that given more time viscoelastic materials will recover further. Thus, although the honeybee silk sponges only recover about 50% of the deformation energy during the cyclic test, if they are allowed one minute relaxation time then they show only a 10% decrease in energy to compress (Fig. 5D). If a sponge is left in solution for 24 hours after a series of compressions its mechanical properties recover fully to those seen in the initial compression. Table 3 includes data for the seventh compression of a 120 minute heat treated sample compressed seven times over a period of 32 days in PBS. Whilst the sample has maintained its integrity and the energy required to compress it remains unchanged, its properties have changed. The sponge has become softer and more resilient after multiple compressions and a lengthy immersion in PBS.

### Mechanism of stabilization in heat treated materials

**Figure 6:**
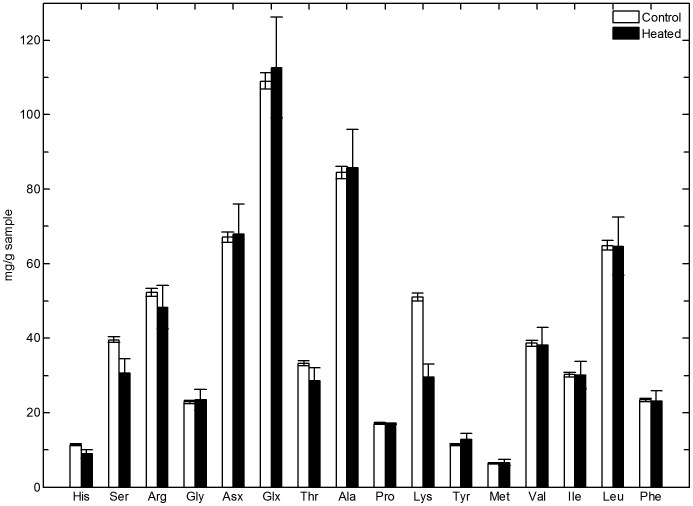
Amino acid analysis results obtained by acid hydrolysis, comparing a control untreated honeybee silk sponge sample to a sponge sample heat treated at 190°C for 60 minutes. Error bars represent one standard deviation.

As described above, the secondary structural changes seen in the heat treated materials are insufficient to explain their physical and mechanical properties. The observed properties strongly suggest covalent cross-linking as the stabilization mechanism. Amino acid analysis was performed on acid hydrolyzed control and heat treated (60 minutes at 190°C) sponges and results are shown in Figure 6. Significantly, heat treatment resulted in losses in lysine and serine. Lysine and serine residues are known to form lysinoalanine cross-links in collagen [16] and other proteins [31] subjected to heat treatment, through dehydration of the serine to form a dehydroalanine electrophile which can then undergo nucleophilic attack by the lysine's primary amine [31]. The analogous reaction with threonine, which also exhibits a significant reduction in heat treated sponges, is known to occur, forming methyl-lysinoalanine. Both lysinoalanine and methyl lysinoalanine are difficult to detect by Raman spectroscopy as the strongest bands, those associated with the symmetric C-N-C stretching vibrations, are expected in the 850 to 900 cm^−1^ region [32] which is already rich [29] in protein skeletal vibrations. No significant spectral changes were observed in this region as a function of thermal treatment.

Heat treatment will also generate isopeptide (amide) [33] or ester bonds between acidic residues such as Asp and Glu in close proximity to either amine-functional (Lys, Arg, His, Asn, Gln), or hydroxyl-functional (Ser, Thr, Tyr) residues, respectively [34]. Isopeptide and ester bonds cannot be inferred following acid hydrolysis as this treatment will hydrolyse all amide and ester bonds, returning the original amine, hydroxyl and carboxylate functions, except in the case where Arg could be thermally converted to a primary amine and form isopeptide bonds. In this event, hydrolysis would, of course, only return the primary amine. Indeed, there does appear to be a slight reduction in Arg content upon heat treatment at 190°C for 60 minutes which may be indicative of this manner of cross-linking having occurred. Isopeptide bonds are difficult to observe directly in the Raman spectra owing to their relatively low abundance compared to the backbone amides which will absorb in the same region [29]. While the carbonyl stretching vibrations of ester groups are expected on the high frequency side of the amide I band, its weak intensity in the Raman compared to that in the infrared [32] makes it hard to detect at low concentrations.

**Figure 7:**
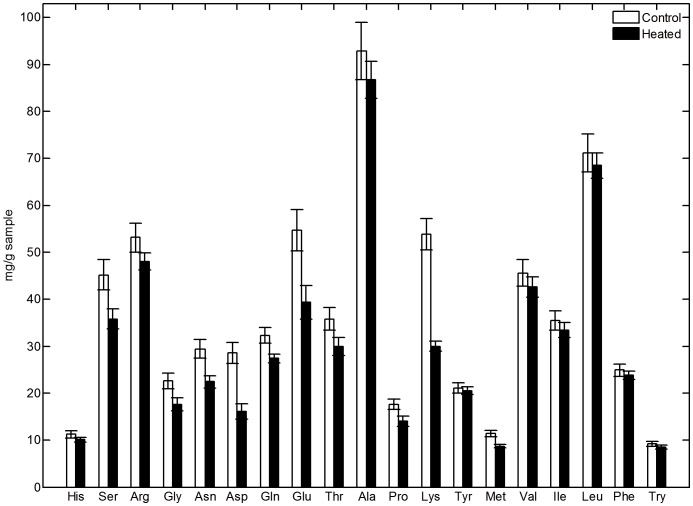
Amino acid analysis results obtained by enzymatic digestion, comparing a control (methanol treated) honeybee silk sponge sample to a methanol treated sponge sample heat treated at 190°C for 60 minutes.

**Figure 8:**
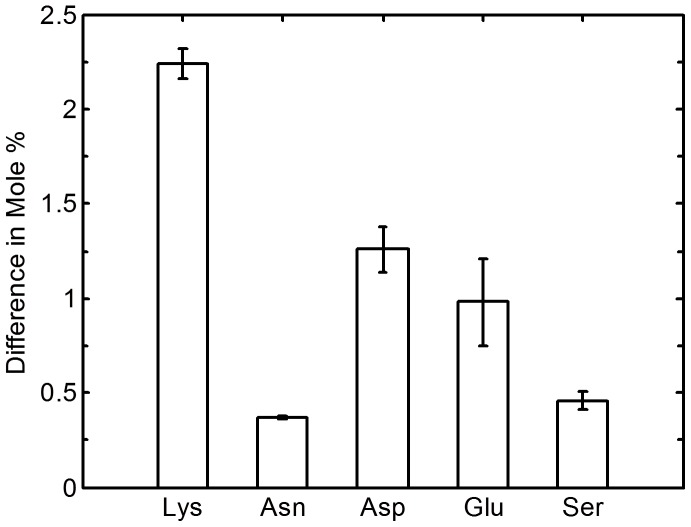
Mole percentage difference for the amino acids lysine, asparagine, aspartic acid, glutamic acid and serine, obtained by enzymatic digestion comparing a control untreated honeybee silk sponge to a sponge sample heat treated at 190°C for 60 minutes.

Therefore, to measure the contribution of isopeptide and ester bonds to the heat treated material's properties, amino acid analysis was conducted on enzymatically hydrolysed control and heat treated (60 minutes at 190°C) sponges (Fig. 7). The enzymes used to hydrolyse the protein's peptide bonds will not cleave isopeptide bonds and comparative reduction in amino acid levels will indicate that the amino acid is involved in cross-linking reactions. The enzymatically hydrolysed heat treated material showed significant losses in lysine, asparagine, aspartic acid and glutamic acid in comparison to the control sponge. The concentrations of histidine and tyrosine were not found to decrease. These differences as well as that observed for serine are represented as mole percentages in Figure 8. These results are consistent with the formation of isopeptide linkages between lysine/asparagine and aspartic acid/glutamic acid as well as the formation of lysinoalanine linkages between lysine and serine and ester linkages between aspartic acid/glutamic acid and serine

**Figure 9:**
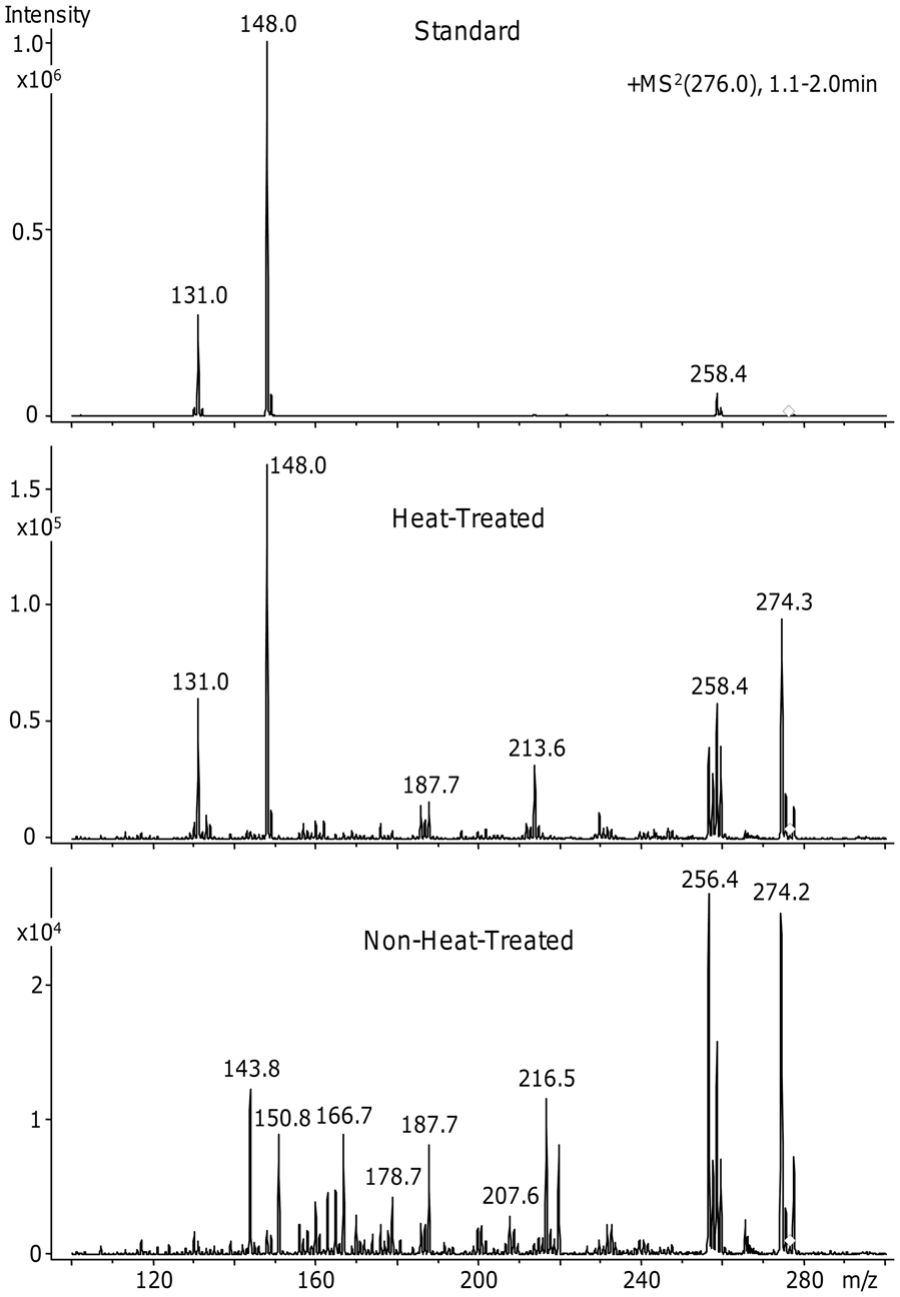
LC-MS/MS detection of the isopeptide crosslink, ε-(γ-glutamyl)-lysine. The panels show tandem mass spectra at 1 to 2 minutes monitoring *m/z* = 276 for the ε-(γ-glutamyl)-lysine standard, for heat-treated, enzyme-digested honeybee silk sponge and for non-heat-treated, enzyme-digested honeybee silk.

Enzymatic hydrolysis of material containing Lys-Glu isopeptide bonds will liberate ε-(γ-glutamyl)-lysine. Targeted LC-MS/MS directly demonstrated the presence of ε-(γ-glutamyl)-lysine in enzymatic digests of heat-treated honeybee silk sponges but not in non-heat-treated sponges (Fig. 9). Following calibration with a ε-(γ-glutamyl)-lysine standard, the amino acid analysis traces were re-examined. A peak corresponding to ε-(γ-glutamyl)-lysine was present in the heat-treated sponge at about 4.4 mg/g sponge corresponding to approximately one ε-(γ-glutamyl)-lysine moiety per protein molecule or two Lys-Glu isopeptide links on average between protein molecules. Heat treatment is also expected to result in analogous isopeptide bonds between lysine and aspartate residues.

## Conclusions

Recombinant honeybee silk proteins can be fabricated into a range of forms that could be used as biomedical materials. The performance of the material can be tailored by stabilization treatments. Aqueous methanol treatment renders the material water insoluble and generates a reversibly compressible sponge. The methanol treatment generates ß-sheets in the material, presumably leading to ß-sheet cross-linking between proteins. The amount of ß-sheet formation can be controlled by varying methanol concentration. Dry heat treatments also render the material water stable with heated samples being stiffer in aqueous solution than methanol treated counterparts. In contrast to the methanol treatment, the changes in physical properties do not correlate with secondary structure molecular changes, with Raman spectra remaining broadly the same. Amino acid analysis on acid digested samples revealed a substantial loss of lysine and marginal losses of serine and threonine in dry heated sponges indicating the possible formation of lysinoalanine and/or methyl-lysinoalanine cross-links, respectively. Amino acid analysis on enzyme digested samples showed an additional loss of asparagine, aspartic acid and glutamic acid, consistent with the formation of isopeptide and ester cross-links as a consequence of heat treatment. The presence of the isopeptide ε-(γ-glutamyl)-lysine was confirmed directly by LC-MS/MS.

The ability to readily control the formation of two distinct cross-linking mechanisms that occur intrinsic to the material is unusual. Whilst silkworm or tussah silk fibroins readily form ß-sheet cross-links, heat treatment is unlikely to result in significant cross-linking because these proteins are poor in the charged residues that can participate in dehydration reactions. Intrinsic covalent cross-links occur in collagen upon heat treatment. However, structural rearrangements are constrained due to the existing native cross-links that stabilize its coiled coil structure. Recombinant honeybee silk proteins are rich in charged residues and contain regions that readily form ß-sheet, so stabilization treatments can cause covalent cross-linking, structural transitions or a combination of the two. In contrast to collagen-based biomedical materials, these honeybee silk proteins can be effectively stabilized without the need for any chemical crosslinking treatments that may leave cytotoxic chemical residues in the sample.
