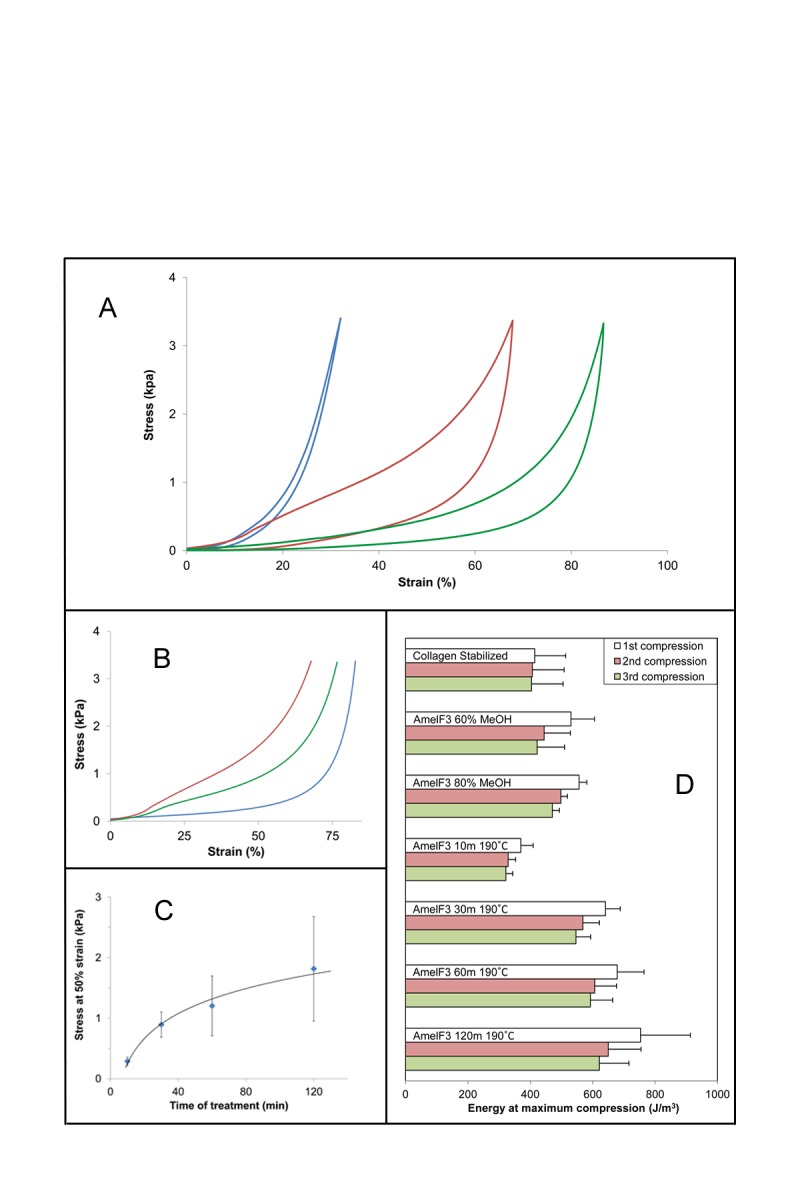# Correction: Controlling the Molecular Structure and Physical Properties of Artificial Honeybee Silk by Heating or by Immersion in Solvents

**DOI:** 10.1371/annotation/2092c30f-6ed4-4c93-a7da-4a0e938b44e9

**Published:** 2014-01-02

**Authors:** Mickey G. Huson, Jeffrey S. Church, Jacinta M. Poole, Sarah Weisman, Alagacone Sriskantha, Andrew C. Warden, Peter M. Campbell, John A. M. Ramshaw, Tara D. Sutherland

The labels for Figure 5D were omitted. Please see the corrected Figure 5 here: 

**Figure:**